# Corticosteroids in Patients with IgA Nephropathy and Severe Chronic Renal Damage

**DOI:** 10.1155/2012/180691

**Published:** 2012-10-10

**Authors:** Claudio Pozzi, Francesca Ferrario, Bianca Visciano, Lucia Del Vecchio

**Affiliations:** ^1^Department of Nephrology and Dialysis, “Bassini” Hospital, Via Gorki 50, 20092 Cinisello Balsamo, Italy; ^2^Department of Nephrology, Dialysis, and Renal Transplant, Alessandro Manzoni Hospital, Via dell'Eremo 9, 23900 Lecco, Italy

## Abstract

Little is known about the utility of treating patients with advanced IgA nephropathy (IgAN). From 2001 to 2005, four patients came to our observation because of serum creatinine higher than 3 mg/dL, proteinuria ranging from 1.8 to 5.1 g/day, and a histological picture of diffuse sclerotic lesions. A corticosteroid course of 12 months was given. Patients were observed for a mean follow up of 84 months. At the end of the steroid course, proteinuria lowered quickly below 1 g/day in two patients, whereas the other two experienced a slower and less persistent decrease of proteinuria. Despite similar lesion severity at renal biopsy, renal function stabilized only in these two ones. In conclusion, these preliminary observations suggest a possible efficacy of corticosteroids in slowing down the progression of renal disease and in postponing the need of dialysis in IgAN patients with stage IV CKD and severe chronic histological lesions.

## 1. Introduction

A number of patients with IgA nephropathy (IgAN) is referred to a nephrologist only at advanced stages of chronic kidney disease (CKD). According to the Italian Register of Dialysis and Transplantation, in 2008 about 900 patients started dialysis because of a glomerulonephritis [1]. Supposing a 20% of patients with a late diagnosis, in Italy nearly 180 patients start dialysis every year because of a late recognized glomerulonephritis, which is often a silent IgAN.

The presence of diffuse chronic lesions at renal biopsy, which is a common finding in these patients, stands against active treatment. However, the histological picture cannot be considered the only factor driving therapeutic choices. Nearly 20 years ago, a “point of no return” was suggested: for creatinine values exceeding 3 mg/dL, IgAN inevitably progresses towards end-stage renal disease (ESRD) [2]. This has been confirmed by other studies [3], further reducing this limit to 2 mg/dL [4]. This, together with the fear of adverse events and the lack of large randomized clinical trial also in IgAN patients with normal or only mildly reduced renal function till the end of nineties, led to some therapeutic nihilism. Consequently, at present little is still known about treatment efficacy in patients with IgAN and advanced CKD.

ACE inhibitors have been proven effective in patients also with advanced CKD [5]. However, these agents neither can prevent patients reaching rapidly ESRD nor can act on immunological mechanisms, which may remain active also in the more advanced stages of the nephropathy.

In 2002 we reported a case of an IgAN patient, whose serum creatinine exceeded the “point of no return”. A 6-month course of corticosteroids halted the progression of renal disease for six years, indicating that sometimes corticosteroids could modify the course of IgAN also in the so-called late-referral patients [6]. In this case series, we report four patients with advanced IgAN at the time of renal biopsy (CKD stage IV). None of these patients was previously aware of having advanced CKD, and the hypothesis of the need of renal replacement therapy in the near future terrified them. So, they asked us to carry out a treatment to avoid or to postpone that need. We evaluated the request and, after obtaining the patients' consent, we gave a corticosteroid course of 12 months, including methylprednisolone 1 g i.v. for three consecutive days at the beginning of months 1, 3, and 5, followed by oral prednisone 0.5 mg/kg every other day for six months, then 0.2 mg/kg every other day for a further 6 months. This treatment schedule is similar to what we used in a randomised clinical trial of IgAN patients with CKD stage III-IV [7]. The patients underwent clinical and biochemical controls every three months to evaluate the clinical course and to recognize possible side effects of corticosteroids. These patients were followed for a mean of 84 months (range 72–108).

## 2. Case Reports

### 2.1. Case  1

**Figure 1:**
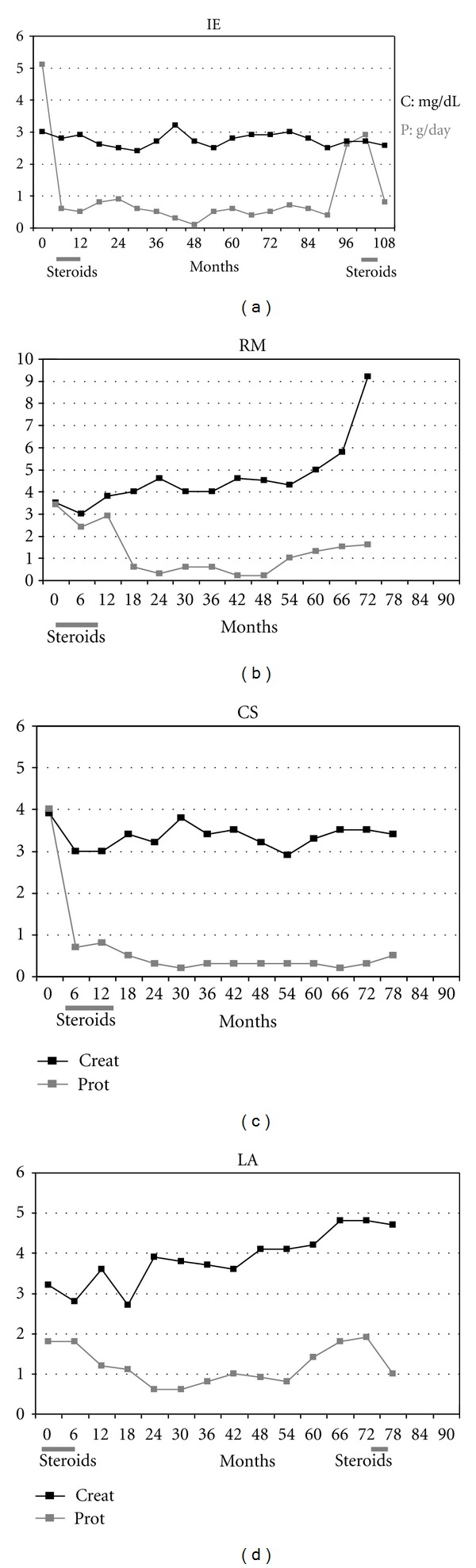
Trend of serum creatinine and proteinuria during the whole follow up in the four cases (evaluated every 6 months).

A 55-year-old man (IE) with a previous history of hypertension treated with candesartan was admitted in our unit in May 2001 for CKD stage IV (serum creatinine of 3.0 mg/dL) and severe proteinuria (5.1 g/day) (Table 1). The patient underwent renal biopsy showing complete glomerular sclerosis in 3 out of 6 glomeruli (50%) and partial sclerosis in other 2 (33%); moderate tubular atrophy and interstitial fibrosis and severe small-artery hyalinosis were observed. Immunofluorescence supported the diagnosis of chronic IgA nephropathy. Corticosteroid were given for one year. Proteinuria rapidly decreased, reaching values of 0.7 g/day after 6 months, and remained below 1 g/day for up to 96 months, when increased again at 2.7 g/day. Because of high proteinuria, six months later a new 6-month course of steroids was performed obtaining a new decrease of proteinuria (0.87 g/day). Renal function remained stable during the whole follow up of 108 months (Figure 1). No important side effects due to corticosteroids were observed. During follow up, the time-average proteinuria was 0.81 ± 0.76 g/day and ΔeGFR was +0.4 mL/min/year; mean blood pressure values were 141/89 mmHg.

### 2.2. Case  2

A 54-year-old man (RM) was admitted to our unit in March 2003 because of CKD stage IV (serum creatinine of 3.5 mg/dL) and proteinuria (3.4 g/day). He was receiving fosinopril because of hypertension lasting 10 years. Baseline clinical characteristics are reported in Table 1. Renal biopsy was performed showing global sclerosis in 5 out of 10 glomeruli (50%) and light mesangial proliferation in the remaining ones; diffuse tubular atrophy and severe interstitial fibrosis together with severe hyalinosis of small arteries were found. According to immunofluorescence findings, a diagnosis of chronic IgA nephropathy was made. The patient received a 12-month steroid therapy without significant side effects. Proteinuria slowly decreased to less than 1 g/day after 18 months but increased again up to 1 g/day after 54 months. Serum creatinine remained stable till month 60, then rapidly increased. Dialysis was started after 72 months of follow up (Figure 1). Time-averaged proteinuria during follow up was 1.1 ± 0.9 g/day; ΔeGFR was −2.3 mL/min/year and mean blood pressure was 136/86 mmHg. 

### 2.3. Case  3

A 46-year-old man (CS) was hospitalized in our unit in February 2004 for CKD stage IV (serum creatinine 3.9 mg/dL) and proteinuria (4.0 g/day). Hypertension had been known since 2001, but enalapril was started only a few weeks before admission. Baseline clinical characteristics are reported in Table 1. At renal biopsy, 6 out of 14 glomeruli (43%) were totally sclerosed; light mesangial proliferation was observed in the remaining 8 ones, with fibroepithelial crescents in 5; diffuse tubular atrophy and severe interstitial fibrosis were also found. Vessels were not evaluable. A diagnosis of chronic IgA nephropathy was made. A corticosteroid course of 12 months was given without side effects. Following treatment, proteinuria rapidly decreased, reaching values less than 1 g/day after 6 months, and remained below 1 g/day for the whole follow up (78 months). Serum creatinine remained stable (3.4 mg/dL at the last observation) (Figure 1). Time-averaged proteinuria was 0.4 ± 0.2 g/day; ΔeGFR was +0.3 mL/min/year, mean blood pressure values were 133/79 mmHg.

### 2.4. Case  4

A 42-year-old man (LA) was admitted to our unit in April 2004 for CKD stage IV (serum creatinine of 3.2 mg/dL); proteinuria and hypertension treated with irbesartan were already known. Baseline clinical characteristics are reported in Table 1. Renal biopsy showed global sclerosis in 4 out of 12 glomeruli (33%); in the remaining 8 glomeruli, mesangial proliferation with segmental sclerosis in 3 was observed; diffuse tubular atrophy, moderate interstitial fibrosis, and initial small artery hyalinosis were found. On immunofluorescence basis, a diagnosis of chronic IgA nephropathy was made. Corticosteroids were given for one year. Proteinuria slowly decreased, reaching values less than 1 g/day after 24 months. At month 42, proteinuria increased again up to 1 g/day. Because of proteinuria persistently >1 g/day, at month 72 months a 6-month steroid course was given again. Proteinuria decreased to 0.97 g/day. Serum creatinine remained stable for 42 months, then increased up to 4.9 mg/dL at month 72. The second corticosteroid course obtained a temporarily halt of progression for the next six months (Figure 1). The overall follow up was of 78 months. Important side effects due to corticosteroids were not observed. Time-averaged proteinuria was 1.1 ± 0.4 g/day, ΔeGFR was −1.4 mL/min/year. Mean blood pressure during follow up was of 130/79 mmHg.

## 3. Discussion

According to the theory of the “point of no return”, patients with IgAN and CKD stage IV, if untreated, start dialysis within one or two years [2, 3].

More than a decade ago, we reported favourable findings without important side effects of a six-month steroid regimen compared with supportive therapy alone in a randomised clinical trial of 86 IgAN patients with proteinuria ≥1 g/dL and serum creatinine ≤1.5 [8].

In these reported cases, our therapeutic decision was driven by our previous favourable experience in patients with more preserved renal function [8] and by the fact that these patients, who had CKD and proteinuria persistently higher than 1 g/day despite inhibition of the renin-angiotensin system (RAS), were at high risk to rapidly progress to ESRD.

In advanced chronic nephropathies, progression seems to be mainly driven by nonimmunological processes. However, histological indexes of activity can also be detected, especially in rapidly progressive patients. Immunosuppressive therapies may potentially reverse proliferative and essudative lesions as much as possible and prevent a further development of glomerular and tubular sclerosis. Indeed, although the pathophysiologic processes ultimately leading to fibrosis are complex, proliferative and essudative lesions could amplify cytokine and growth factor cascades enhancing interstitial infiltration and fibroblast activity. This may be partially broken off by immunosuppressive and/or anti-inflammatory regimens.

Following treatment, renal function and proteinuria behaved differently in the single patients of these case reports. At the end of the steroid course, proteinuria lowered quickly below 1 g/day in two patients, whereas the remaining two experienced a slower and less persistent decrease of proteinuria. This entailed a time-averaged proteinuria below 1 g/day only in the two responder patients. Despite similar lesion severity at renal biopsy, renal function stabilized only in these two ones. Therefore, a decrease of proteinuria below 1 g/day at the end of the therapy and a time averaged proteinuria less than 1 g/day seemed to be associated with a better outcome. Conversely, proteinuria increase over 1 g/day during follow up preceded a further worsening of renal function. These data are in line with the concept that the lower proteinuria is during follow up, the better the outcome is [9, 10]. However, for the first time they suggest that this prognostic factor is of value also in patients with advanced IgAN and that the rate of proteinuria decrease following treatment may be of importance as well.

**Figure 2:**
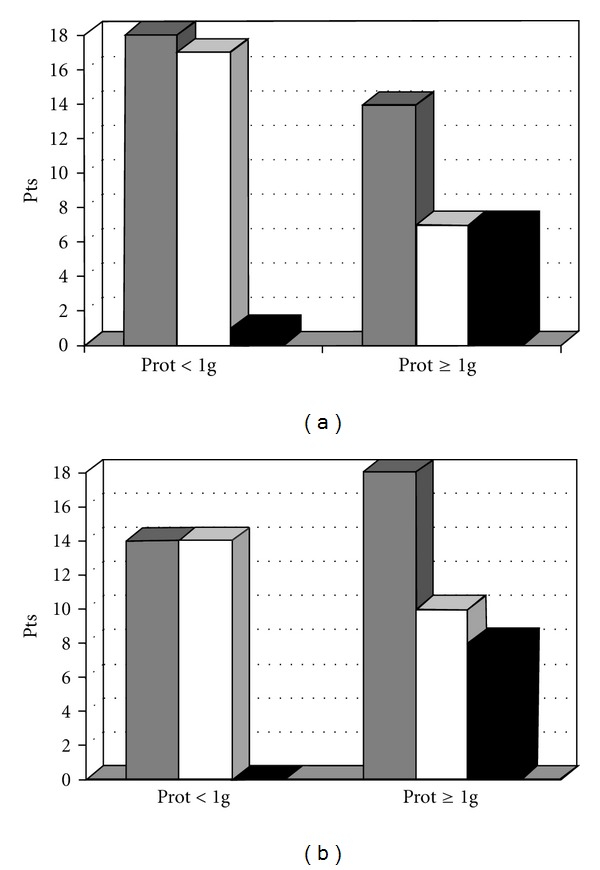
(a) Number of patients with proteinuria either less or ≥1 g/day after 12 months of treatment (grey) and number of patients who either reached (black) or did not reach (white) ESRD after a mean follow up of 48 months [7]. (b) Num]ber of patients with a time-average proteinuria either less or ≥1 g/day (grey), and number of patients who reached (black) or did not reach (white) ESRD after a mean follow up of 48 months [7].

To confirm these preliminary observations, we evaluated the relationship between proteinuria behaviour following treatment and CKD progression in 38 IgAN patients with stage III-IV CKD having adequate follow up (more than 48 months), who were enrolled in a multicentre, clinical trial comparing steroids plus azathioprine to steroids alone and followed up for at least 48 months [7]. Among the eight patients who reached ESRD in the first 48 months, only one had proteinuria below 1 g/day following treatment. Conversely, the majority of the patients who did not reach ESRD (17 out of 24) had a decrease of proteinuria below 1 g/day during follow up. Similarly, the time-averaged proteinuria was ≥1 g/day in all the 8 patients who reached ESRD, while it was <1 g/day in 14 out of 24 patients (58%) who did not reach it (Figure 2). In agreement with our observations, among 47 Japanese patients followed up for a mean of 103 months, none of those with a time-averaged proteinuria <1 g/day progressed to ESRD [4].

Despite the lack of a control group and the small number of this case series, these data suggest that steroid therapy may slow down progression of CKD also in patients with advanced IgAN. In the nineties, Schöll et al. [3] evaluated retrospectively the natural history of IgAN in 115 patients from the “German Glomerulonephritis Therapy Study” and found that after exceeding serum creatinine values of 3 mg/dL, CKD inevitably progressed with serum creatinine level doubling on average from 3 to 6 mg/dL within 10 months. Differing from these findings, none of our 4 patients doubled their serum creatinine during the first 60 months of follow up, and had a much slower progression rate (annual change of estimated GFR, calculated using the MDRD formula [11], between +0.6 and −2.3 mL/min/year).

Our preliminary results are in line with two studies comparing the efficacy of ACE-I alone versus ACE-I plus corticosteroids demonstrating that the addition of steroids resulted in a better renal outcome and in a more potent antiproteinuric effect [12, 13]. However, differing from this case series, the enrolled patients had a normal or a slightly reduced renal function.

Only a few studies have evaluated immunosuppressive therapy in IgAN patients with CKD stage III-IV. The majority were retrospective and enrolled patients with more preserved renal function than our case series, allowing the use of more aggressive regimens including azathioprine, cyclophosphamide, or mycophenolate mofetil (MMF). Goumenos et al. [14] retrospectively evaluated a heterogeneous population of 114 IgAN patients with various degree of CKD (nearly half of the patients had serum creatinine levels of >1.24 mg/dL) and found that a higher percentage of patients receiving steroids plus azathioprine had stable renal function over a follow up of 46 months compared to those receiving only supportive therapy. However, the same authors were not able to confirm their positive findings in another retrospective study of 74 patients observed for a longer follow up period [15]. Mitsuiki et al. [16] reported retrospectively about 35 patients with histologically advanced IgAN and CKD (mean serum creatinine around 2 mg/dL) who were either treated with prednisolone for more than two years and cyclophosphamide for six months (*n* = 27) or received supportive treatment alone (*n* = 8). After five years, all of the patients in the control group had developed ESRD, whereas the majority of the treated patients were still free of dialysis (mean serum creatinine of 4 mg/dL). Treatment-related side effects were observed in only two patients. Ballardie and Roberts [17] performed a prospective, randomized study of 38 patients with progressive IgAN who received prednisolone 40 mg/day (reduced to 10 mg/day after two years) with cyclophosphamide 1.5 mg/kg/day for the first three months followed by the same dose of azathioprine for a minimum of two years, or supportive therapy alone. The treated patients experienced significantly better renal survival and a greater reduction in proteinuria levels than those in the control group. More recently, Roccatello et al. [18] reported a case series of IgAN patients with acute inflammatory histologic changes (diffuse mesangial proliferation with at least 10% florid crescents), CKD (mean serum creatinine 1.6 mg/dL, range 1.2–2.9), and proteinuria (mean 2.4 g/day, range 1.13–5.25) who received a combined schedule of steroids and MMF. Following treatment, serum creatinine and proteinuria significantly dropped at 6 months compared with baseline values and remained lower during a mean follow up of 51 months (range 24–90).

Altogether, these studies suggest that immunosuppressive treatment gives a certain degree of clinical benefit in IgAN patients with CKD stage III-IV. However, none of them compared cytoxic agents plus steroids to steroids alone. According to the findings of our recent trial [7], in CKD patients stage III-IV a small likelihood of reducing the risk of CKD progression with steroids plus azathioprine compared to steroids alone should be well balanced with an increased risk of side effect with the adding of azathioprine to treatment. 

Finally, would the pathological features of our patients been able to predict their renal outcome and treatment response? The answer is no. Traditional classifications of IgAN do not offer enough information to decide which patient deserves treatment [19–24]. Recently, a new pathological classification of IgA nephropathy has been proposed, but it excluded patients with eGFR <30 mL/min [25]. In addition, all the four renal biopsies showing advanced damage with a prevalence of diffuse chronic lesions were against a possible usefulness of corticosteroids. 

In conclusion, these preliminary observations suggest a possible efficacy of corticosteroids in slowing down the progression of the renal disease and in postponing the need of dialysis in IgAN patients with stage IV CKD and severe chronic histological lesions. 

## Figures and Tables

**Table 1:** Baseline clinical and histological characteristics of the four patients.

Patient	IE	RM	CS	LA
Age (years)	55	54	46	42
Body mass index (kg/m^2^)	31	26	28	27
Systolic BP (mmHg)	162	155	130	123
Diastolic BP (mmHg)	92	90	80	80
Serum creatinine (mg/dL)	3.0	3.5	3.9	3.2
Proteinuria (g/day)	5.1	3.4	4.0	1.8
eGFR (using MDRD)	26.2	19.5	17.8	22.7
Treatment with ACEI	No	Yes	Yes	No
Treatment with ARB	Yes	No	No	Yes
Glomeruli with global sclerosis	50%	50%	43%	33%
Tubular atrophy	25–50%	>50%	>50%	25–50%
Interstitial fibrosis	Moderate	Severe	Severe	Moderate
Arterial sclerosis	Severe	Severe	Not Eval.	Moderate
